# Glucocorticoids coordinate macrophage metabolism through the regulation of the tricarboxylic acid cycle

**DOI:** 10.1016/j.molmet.2021.101424

**Published:** 2021-12-22

**Authors:** Ulrich Stifel, Eva-Maria Wolfschmitt, Josef Vogt, Ulrich Wachter, Sabine Vettorazzi, Daniel Tews, Melanie Hogg, Fabian Zink, Nora Maria Koll, Sandra Winning, Rémi Mounier, Bénédicte Chazaud, Peter Radermacher, Pamela Fischer-Posovszky, Giorgio Caratti, Jan Tuckermann

**Affiliations:** 1 Institute of Comparative Molecular Endocrinology (CME), Ulm University, Ulm, Germany; 2 Institute for Anesthesiological Pathophysiology and Process Engineering, and Department of Anesthesiology, University Hospital, Ulm, Germany; 3 Division of Pediatric Endocrinology and Diabetes, Department of Pediatric and Adolescent Medicine, Ulm University Medical Center, Ulm, Germany; 4 Institut fürPhysiologie, Universitätsklinikum Essen, Universität Duisburg-Essen, 45122, Essen, Germany; 5 Institut NeuroMyoGène, Université Claude Bernard Lyon 1, CNRS UMR 5310, INSERM U1217, Université Lyon, Lyon, France; 6 Department of Pediatric and Adolescent Medicine, Ulm University Medical Center, Ulm, Germany

**Keywords:** Immunometabolism, TCA Cycle, Macrophage, Glucocorticoids, Succinate

## Abstract

**Objectives:**

Glucocorticoids (GCs) are one of the most widely prescribed anti-inflammatory drugs. By acting through their cognate receptor, the glucocorticoid receptor (GR), GCs downregulate the expression of pro-inflammatory genes and upregulate the expression of anti-inflammatory genes. Metabolic pathways have recently been identified as key parts of both the inflammatory activation and anti-inflammatory polarization of macrophages, immune cells responsible for acute inflammation and tissue repair. It is currently unknown whether GCs control macrophage metabolism, and if so, to what extent metabolic regulation by GCs confers anti-inflammatory activity.

**Methods:**

Using transcriptomic and metabolomic profiling of macrophages, we identified GC-controlled pathways involved in metabolism, especially in mitochondrial function.

**Results:**

Metabolic analyses revealed that GCs repress glycolysis in inflammatory myeloid cells and promote tricarboxylic acid (TCA) cycle flux, promoting succinate metabolism and preventing intracellular accumulation of succinate. Inhibition of ATP synthase attenuated GC-induced transcriptional changes, likely through stalling of TCA cycle anaplerosis. We further identified a glycolytic regulatory transcription factor, HIF1α, as regulated by GCs, and as a key regulator of GC responsiveness during inflammatory challenge.

**Conclusions:**

Our findings link metabolism to gene regulation by GCs in macrophages.

## Highlights

• Glucocorticoids regulate a network of genes associated with cellular metabolism in macrophages.
• Glucocorticoids affect the macrophage metabolome, converging on reactive oxygen species and tricarboxylic acid.
• Glucocorticoids mediate Tricarboxylic acid cycle anaplerosis at the glutamine-α-ketoglutarate pathway and this is required for anti-inflammatory gene regulation by glucocorticoids.
• Glucocorticoids prevent Lipopolysaccharide-induced succinate accumulation, and HIF1α stabilization.

## 1 Introduction

Glucocorticoids (GCs) are among the most potent anti-inflammatory agents known [1]. Since their discovery in the 1950s, GCs are still used by clinicians to treat various inflammatory disorders, such as rheumatoid arthritis, lupus, sepsis, and acute lung injury associated with SARS-COV-2 infection [[2], [3], [4], [5]]. Innate immune cells, such as macrophages, are a major target of GC therapy [6], but the mechanisms by which GCs inhibit the inflammatory activation in macrophages are still not fully understood.

GCs signal through the glucocorticoid receptor (GR), a ligand-activated transcription factor, which regulates gene expression. GR activates various anti-inflammatory genes such as *Gilz* (*Tsc22d3*) and *Dusp1* [7,8] while repressing the expression of pro-inflammatory cytokines, e.g. *Tnf* [9]. However, merely suppressing cytokine production alone is not sufficient for the anti-inflammatory actions of GR, suggesting other important mechanisms at play. We and others have proposed the induction of anti-inflammatory genes as a key aspect of GR suppression of inflammation [[10], [11], [12], [13], [14]]; however, whether GCs regulate macrophage metabolism has been overlooked.

Although it is well documented that GCs control the metabolic functions of various tissues (e.g. liver and adipose [15, 16]), and there are reports of GCs regulating mitochondrial function in neuronal cells [17, 18], the contribution of GCs to the cellular metabolism of macrophages remains an intriguing question. Metabolic regulation in macrophages has recently become an area of interest due to metabolic rewiring driving both pro- and anti-inflammatory processes [[19], [20], [21]]. Activation of pro-inflammatory macrophages results in an increase in glycolysis, pentose-phosphate shunt, and mitochondrial dysfunction [22],while treatment with anti-inflammatory cytokines such as IL-4 promotes mitochondrial functionality and network connectivity and the switch to more oxidative metabolism [22]. Inflammatory signals influence mitochondrial function, leading to increased ROS production and the accumulation of tricarboxylic acid cycle (TCA) intermediates, which have downstream effects on macrophage function. For example, succinate stabilizes HIF1α expression, while itaconate acts as a negative feedback loop on inflammation [23,24].

Here, we analyzed the macrophage transcriptome and metabolome in response to lipopolysaccharide (LPS) and GC treatment *in vitro*. We identified the pathways involved in TCA cycle functionality under control of GCs, which prevent the accumulation of succinate and reactive oxygen species. Inhibition of mitochondrial functionality or addition of exogenous succinate inhibits GC transcriptional actions through a HIF1α-GR regulatory axis.

## 2 Results

### 2.1 Glucocorticoids regulate genes involved in cellular metabolism

**Figure 1:**
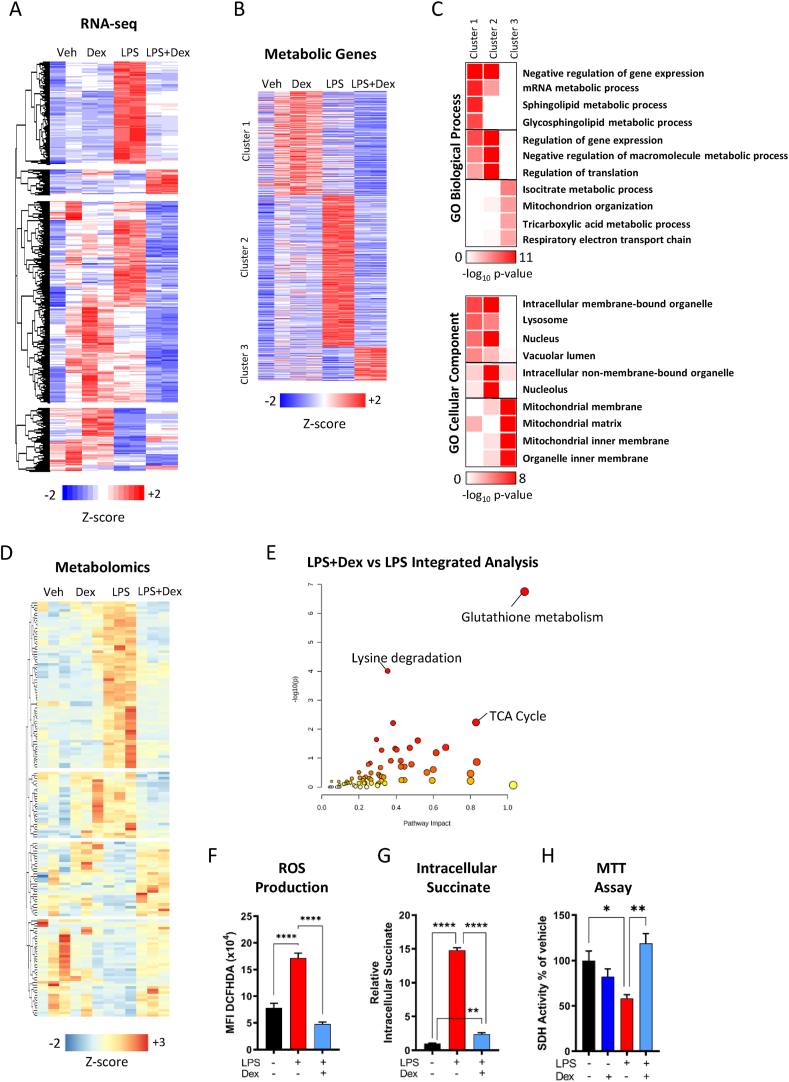
**Glucocorticoids Regulate Macrophage Metabolic Genes**. (A) Hierarchical clustering of RNA seq in vehicle, Dex (100 nM), LPS (100 ng/ml), or LPS + Dex 24-h-treated macrophages based on differentially regulated genes between LPS and LPS + Dex (N = 2). (B) Clustering of genes associated with metabolism (N = 3). (C) Gene ontology analysis of clusters from B. (D) Hierarchical clustering of metabolites identified in vehicle, Dex (100 nM), LPS (100 ng/ml), or LPS + Dex 24-h-treated macrophages by untargeted metabolomics based on differentially regulated metabolites between LPS and LPS + Dex. (E) Integrative KEGG pathway analysis of differently regulated metabolites and differentially regulated genes from A. (F) 2′,7′-Dichlorfluorescein-Diacetat (DCFHDA) fluorescence of macrophages treated with vehicle, LPS, or LPS + Dex for 16 h measured on a plate reader (N = 5). (G) Intracellular succinate accumulation of macrophage treated with vehicle, LPS, or LPS + Dex for 24 h (N = 6). (H) SDH activity was measured using MTT in macrophages treated with vehicle, LPS, or LPS + Dex for 24 h (N = 5). Data are presented as z-scores, -log10 p-value, or mean + SEM. Statistical analysis was performed by one-way ANOVA using a Tukey post-hoc test. P < 0.05∗, <0.01 ∗∗, <0.001∗∗∗, <0.0001 ∗∗∗∗.j

In order to assess whether GCs potentially regulate the metabolic pathways under inflammatory conditions, we analyzed RNA-seq from bone marrow-derived macrophages (BMDMs) treated with vehicle (DMSO), dexamethasone (Dex, 100 nM), LPS (lipopolysaccharide, 100 ng/ml) or LPS + dex for 24 h. Genes differentially regulated (p < 0.05) between LPS- and LPS + Dex-treated samples were used for clustering (Figure 1A, Fig. S1A), and the resulting differentially expressed genes were assessed for gene ontology enrichment (Fig. S1B).

We identified multiple pathways involved in metabolic functionality enriched in gene sets upregulated and downregulated in LPS + Dex versus LPS conditions. Ontological terms linked to neutrophil biology and the electron transport chain were highly enriched in upregulated genes. To investigate this further, all genes involved in an ontology related to metabolism were manually selected and clustered using K-means clustering (Figure 1B). Subsequently, individual clusters were reanalyzed by gene ontology to determine which metabolic pathways were altered in each cluster (Figure 1C). We identified three main clusters, consisting of genes upregulated by LPS + Dex (cluster 3), genes upregulated by LPS and repressed by the addition of Dex (cluster 2), and a less well-defined cluster with genes upregulated by the addition of Dex (cluster 1) (Supplementary Table 1). Cluster 3 exhibited enrichment for genes involved in the TCA cycle and the function and development of mitochondria. The GO cellular component also localized these genes to the mitochondria. Cluster 2 was highly enriched for the negative regulation of gene expression, as well as translation. Cluster 1 was enriched for the regulation of gene expression and lipid metabolites, such as sphingolipids or glycosphingolipids, consistent with our previous reports [13]. Both clusters 1 and 2 were enriched for localization to the nucleus or non-mitochondrial organelles. In addition, we validated genes from all three clusters via qPCR (Fig. S1C). Based on these gene expression data, we hypothesized that GC control of metabolism may be important for anti-inflammatory actions in macrophages.

### 2.2 Metabolomics identifies tricarboxylic acid cycle and reactive oxygen species as glucocorticoid regulated

To better understand how the identified GC-regulated genes involved in macrophage metabolism have consequences for metabolite abundance, we performed unbiased metabolomics. By this, we aimed to identify the metabolites regulated by GCs in macrophages and thus infer pathways. After 24-h treatment, the cells were analyzed by LC/MS. Metabolites, with a KEGG identifier, were clustered using hierarchical clustering based on differential detection in LPS vs LPS + Dex (p < 0.1) (Figure 1D, Supplementary Table 2). While LPS and Dex alone induced specific changes in the metabolome of macrophages, the co-treatment of LPS + Dex returned the detected metabolites back to a state similar to that of vehicle-treated cells (Fig. S2A). The pathways involved in amino acid metabolism as well as glutamine and glutathione metabolism were highly enriched in the metabolites differentially detected between LPS and LPS + Dex (Fig. S2B). However, integration of the differentially expressed genes from Figure 1A with the metabolomics using MetaboAnalyst-based KEGG pathway analysis resulted in the enrichment of three clear pathways (Figure 1E, Supplementary Table 3). While each pathway has an important functionality individually, glutathione metabolism, TCA cycle, and lysine metabolism are all linked to the regulation of reactive oxygen species (ROS) [[25], [26], [27]]. Indeed, LPS-induced ROS production was inhibited by the treatment of macrophages with GCs (Figure 1F). These data collectively suggest that GC regulation of metabolism has functional consequences for macrophages. However, the enrichment of the TCA cycle pathway in the integrative analysis was of interest due to the knowledge that TCA cycle intermediates can play important roles in macrophage action [28]. Indeed, GCs treatment of macrophages suppressed the LPS-induced induction of intracellular succinate accumulation (Figure 1G), suggesting a clear role of GCs in regulating mitochondrial and TCA cycle functionality. Furthermore, the activity of succinate dehydrogenase (SDH) was strongly impaired after LPS treatment, which was recovered by the addition of Dex (Figure 1H)

### 2.3 The mitochondrial network, but not oxidative metabolism, is regulated by glucocorticoids

**Figure 2:**
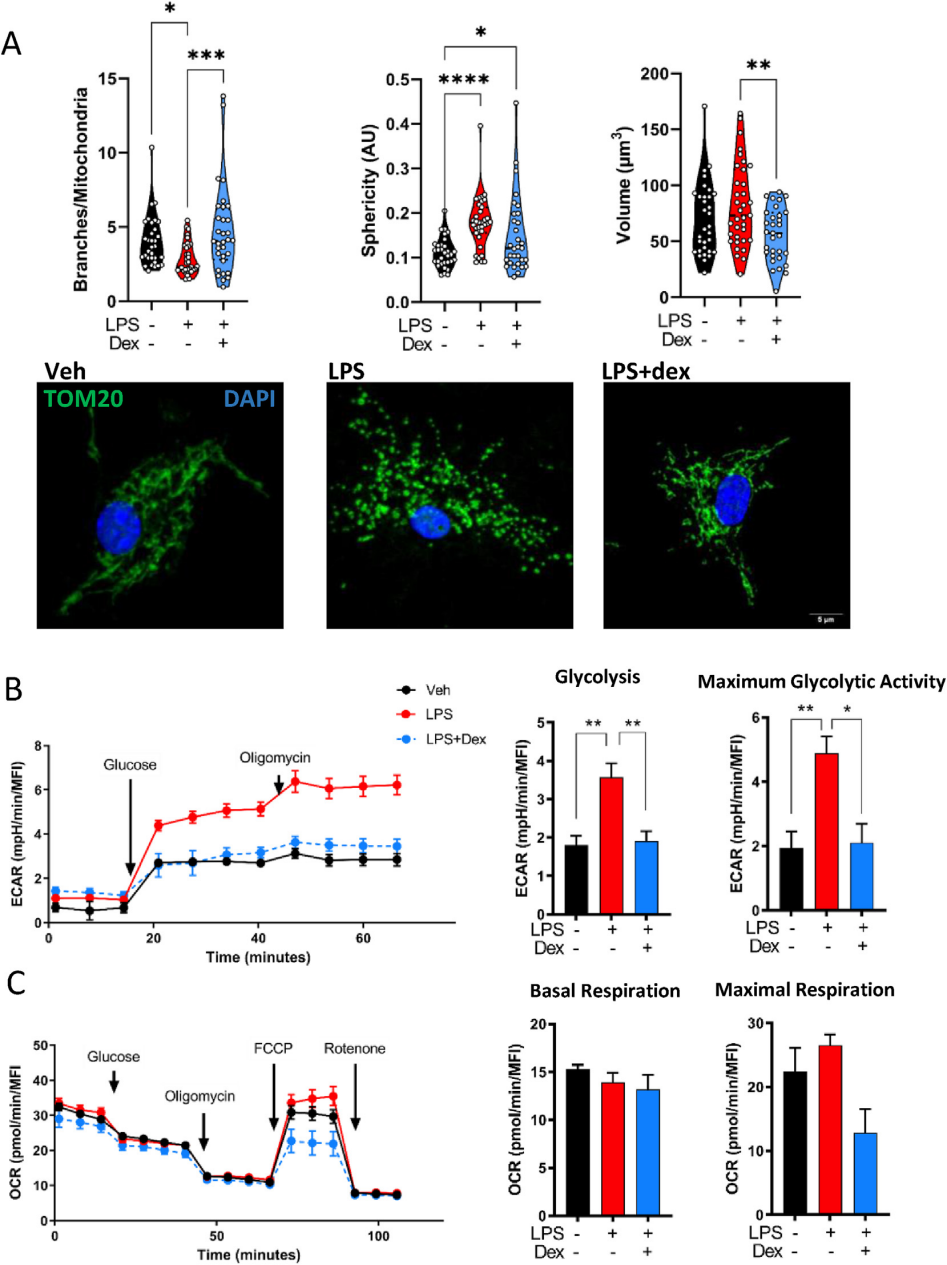
**Mitochondrial Dynamics are Glucocorticoid Responsive**. (A) Macrophages were treated for 24 h with vehicle, LPS (100 ng/ml), or LPS + Dex (100 nM) before analysis. Immunofluorescence images of mitochondria (anti-TOM20) were quantified by Mitochondria Analyzer ImageJ plug-in to determine mitochondrial morphology. Each data point represents an individual cell (N = 31–35). Data from 4 separate macrophage isolations. (B, C) Metabolic flux in macrophages was analyzed using the Seahorse extracellular flux analyzer. Cells were treated for 24 h with vehicle, Dex, (100 nM), LPS (10 ng/ml), or a combination of LPS + Dex before measurement. Data normalized to well DNA content. N = 3. Scale bar: 5 μm (A). Data are presented as violin plots. Statistical analysis was performed by one-way ANOVA using a Tukey post-hoc test. p < 0.05∗, <0.01 ∗∗, <0.001∗∗∗, <0.0001 ∗∗∗∗.

Next, we analyzed mitochondrial morphology in macrophages by the immunofluorescence of the mitochondrial marker TOM20 (Figure 2A). LPS induced the fragmentation of mitochondria, as demonstrated by the reduced number of branches per mitochondrion. This coincided with increased sphericity of each mitochondrion [29]. These changes were not accompanied by differences in the mitochondrial volume, indicating a reduction in mitochondrial interconnectivity, rather than an increase in mitochondrial biogenesis. GC treatment partially recovered the fragmentary effect of LPS on the mitochondrial network, increasing branching and demonstrating the protective effect of Dex on the mitochondria under inflammatory conditions. However, GCs did reduce the mitochondrial volume, in comparison with vehicle and LPS treatments. In turn, we investigated the mitochondrial copy number by measuring the expression of mitochondrial DNA relative to genomic DNA but could not observe any changes in response to LPS + Dex (Fig. S2C), which indicates that multiple levels of control are involved in the determination of the mitochondrial number and volume.

To test whether the effects detected by transcriptomics, metabolomics, and mitochondrial morphology translated into differences in glycolytic or oxidative metabolism, we analyzed metabolism in real time using a Seahorse Extracellular Flux analyzer in two myeloid cell types, macrophages, and bone marrow-derived dendritic cells (BMDCs), known to have metabolism altered by inflammatory stimuli [32]. In macrophages, LPS caused an increase in the extracellular acidification rate (ECAR), and maximal ECAR, which was repressed to baseline by GCs (Figure 2B), indicating direct control of GCs on glycolytic activity in macrophages. Surprisingly, despite our findings on the TCA cycle through metabolomics and mitochondrial morphology, we did not observe any effect of GCs on oxygen consumption, neither basal nor maximal, despite a trend toward decreased maximal oxygen consumption (Figure 2C). We did not observe this with the treatment with 10 ng/μl or 100 ng/μl LPS, while the effect of ECAR was consistent within both treatments (Figure 2B, Fig. S3C). In dendritic cells, the combination of LPS + Dex suppressed glycolysis to a statistically significant extent similarly to macrophages, but both Dex or LPS + Dex inhibited maximal glycolytic activity in contrast with macrophages (Fig. S3A). Furthermore, LPS increased respiration, which was inhibited by the further addition of GCs, while maximal respiration was inhibited by all treatments (Fig. S3B). These data indicate that GCs have the ability to inhibit metabolism in two different myeloid cell types, albeit in different ways

### 2.4 Glucocorticoids promote tricarboxylic acid cycle anaplerosis

**Figure 3:**
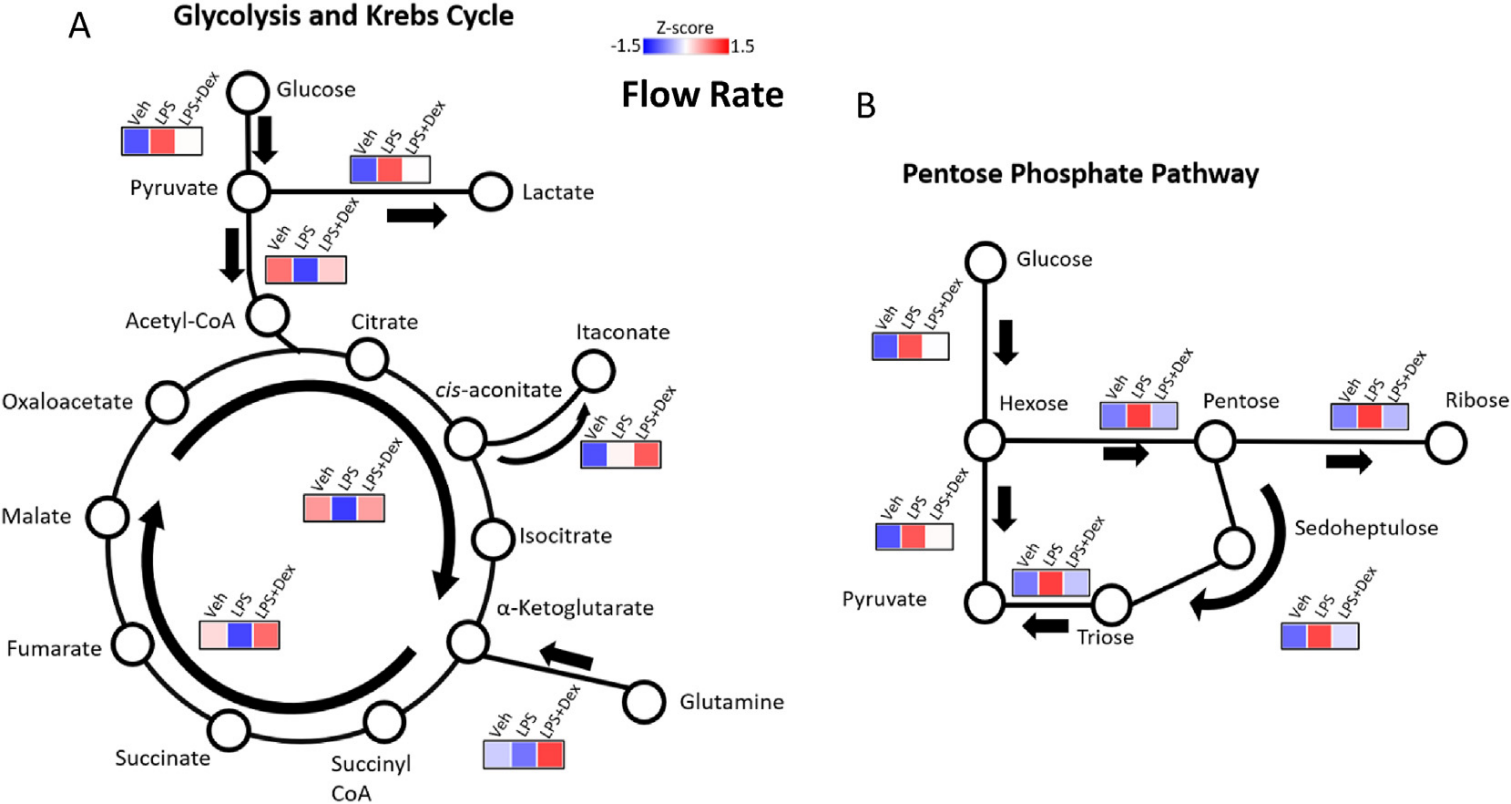
**TCA Cycle Anaplerosis is Promoted by Glucocorticoids**. Metabolic flux through (A) the glycolytic pathway, tricarboxylic acid pathway, and (B) pentose phosphate pathway was analyzed by GC/MS after macrophages were treated for 24 h with vehicle, LPS (100 ng/ml), or LPS + Dex (100 nM) and incubated with labeled glucose and glutamine. Cells from 7 mice were combined into two replicates. Data were presented as z-scores and graphical representation of the pathways.

The identification of the TCA cycle as a highly enriched pathway through integrative metabolomics and transcriptomics, as well as changes in the mitochondrial morphology led us to hypothesize that GCs play a role in the regulation of TCA cycle intermediates but not oxidative phosphorylation. We therefore used high-resolution metabolic flux analysis to determine the flow rate of stable isotope-labelled glucose and glutamine through the TCA cycle in vehicle-, LPS-, and LPS + Dex-treated macrophages using gas chromatography followed by mass spectrometry (GC/MS). LPS promoted glucose conversion into lactate and entry into the pentose phosphate pathway (Figure 3A,B), a pathway known to be involved in ROS production [38]. LPS also inhibited glutamine uptake and conversion into α-ketoglutarate; however, the addition of GCs increased glutamine metabolism (Figure 3A), consistent with the generation of the anti-inflammatory macrophage phenotype [30]. Furthermore, TCA cycle anaplerosis was inhibited by LPS via the glutamine to the α-ketoglutarate pathway, but this was overcompensated by GCs, further increasing flux. The GC-dependent effects on the TCA cycle matched with our observation that LPS-driven cytoplasmic succinate accumulation was repressed by Dex (Figure 1G), likely due to the increased flow rate through the TCA cycle, consuming succinate.

### 2.5 Activation of the glucocorticoid receptor in macrophages promotes mitochondrial Network and TCA cycle genes in vivo

**Figure 4:**
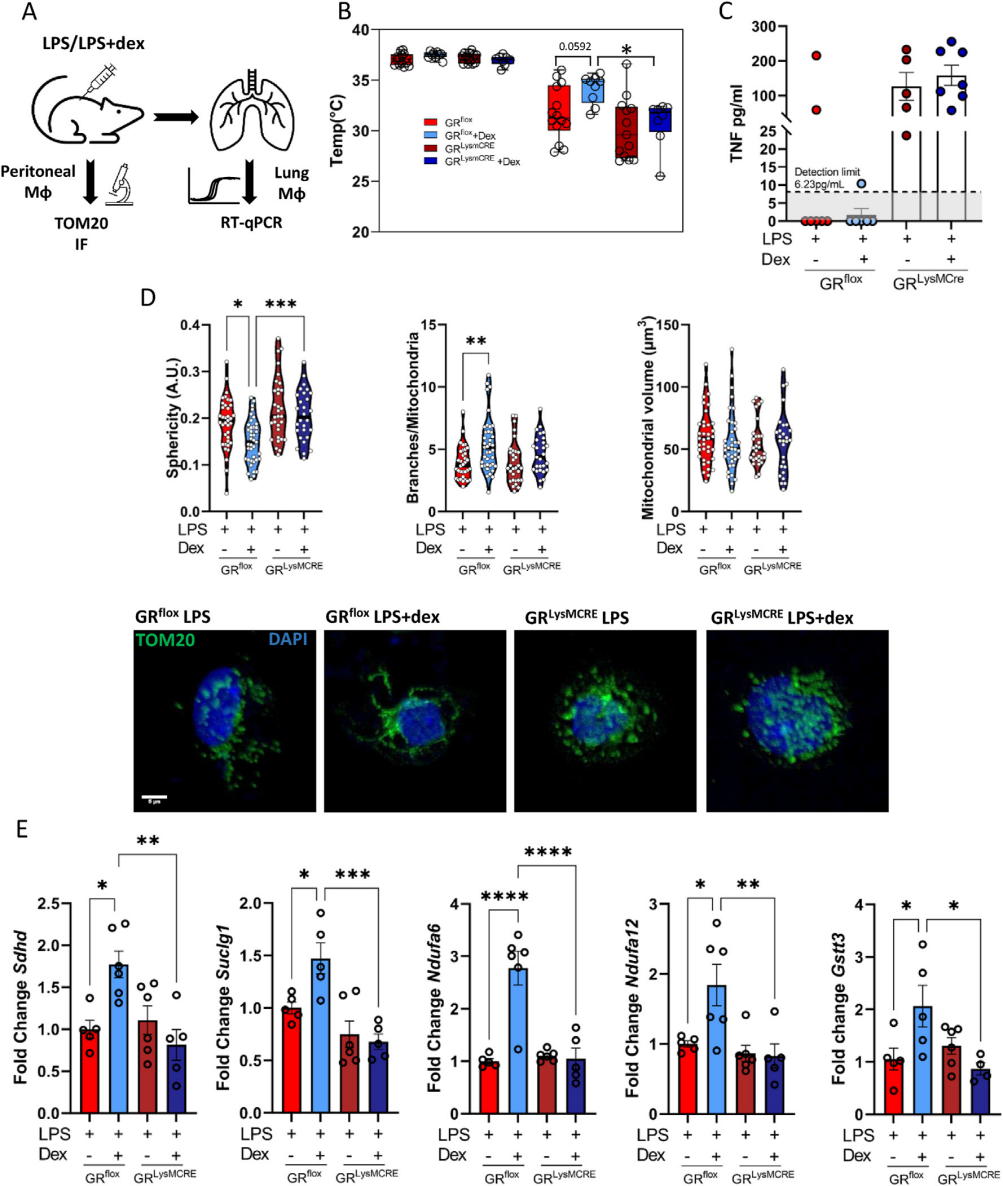
Activation of the Glucocorticoid Receptor Regulates Macrophage Metabolism In Vivo. GR^flox^ and GR^LysMCRE^ mice were subjected to experimental endotoxin shock for 24 h with or without Dex treatment. (A) Schematic of experiment and cell isolations. (B) Rectal temperature of mice before LPS and 24 h after LPS treatment (N = 8–14). (C) Serum TNF was determined by ELISA (N = 5–7). (D) Immunofluorescence images of mitochondria (anti-TOM20) from peritoneal macrophages were quantified by Mitochondria Analyzer ImageJ plug-in to determine mitochondrial morphology. Each data point represents an individual cell (N = 28–39) from 6 to 7 mice per group. (E) Lung macrophages were isolated, and gene expression was determined by qPCR (N = 5–6). Data are presented as mean with individual datapoints representing a mouse (B, C, E), or a cell (D). Data represent 4 independent experiments. Statistical analysis was performed by 2-way ANOVA (B) or one-way ANOVA (D, E). p < 0.05∗, <0.01∗∗, <0.001∗∗∗, <0.0001∗∗∗∗.

We next aimed to assess whether the activation of GR in a mouse model of endotoxin shock would also result in changes in the macrophage mitochondrial function. To do so, we treated wildtype (GR^flox^) mice or mice with a deletion of GR in the macrophage/myeloid lineage (GR^LysMCRE^) with an intraperitoneal dose of LPS or a combination of LPS and Dex. After 24 h, two distinct macrophage populations were isolated from the same mice and were analyzed separately due to material limitations. Lung macrophages were isolated using preparative sorting (Fig. S4A) and purity was confirmed by qPCR (Fig. S4B). Peritoneal macrophages were isolated and selected via adherence enrichment (Figure 4A). Treatment with Dex prevented the dramatic loss in body temperature, but only in GR^flox^ mice (Figure 4B), demonstrating the contribution of macrophage GR to disease tolerance. There was no effect on weight loss in the groups during the 24-h time course (Fig. S4C). Loss of macrophage GR resulted in increased TNF in the serum of mice after 24 h with no response to Dex (Figure 4C). To determine the role of metabolic regulation by GCs in macrophages, we initially assessed the mitochondrial network of peritoneal macrophages. Indeed, reminiscent to the *in vitro* data in Figure 2, the mitochondrial network was highly fragmented under LPS conditions, in both GR^flox^ and GR^LysMCRE^ macrophages. Dex, however, reduced the sphericity of mitochondria and increased the branches per mitochondrion, which did not occur in the GR-deficient macrophages (Figure 4D), reestablishing the deleterious mitochondrial network that occurs after LPS treatment. However, in contrast with the *in vitro* experiments, there was no effect on the mitochondrial volume in macrophages isolated *ex vivo*. To validate our transcriptomic analysis and integrative analysis (Figure 1B,E), macrophages isolated from the lungs of LPS- and LPS + Dex-treated mice were analyzed by RT-qPCR. Genes involved in succinate metabolism, *Sdhd* and *Suclg1*, as well as in mitochondrial NADH metabolism, *Ndufa6* and *Ndufa12*, were all upregulated by Dex in macrophages isolated from wildtype mice but not in those lacking GR (Figure 4E). We also found*Gstt3*, highlighted in the integrative analysis as a regulator of GST metabolism, and therefore ROS was activated in Dex-treated GR^flox^ macrophages (Figure 4E).

### 2.6 Mitochondrial function is required for glucocorticoid-mediated gene expression

**Figure 5:**
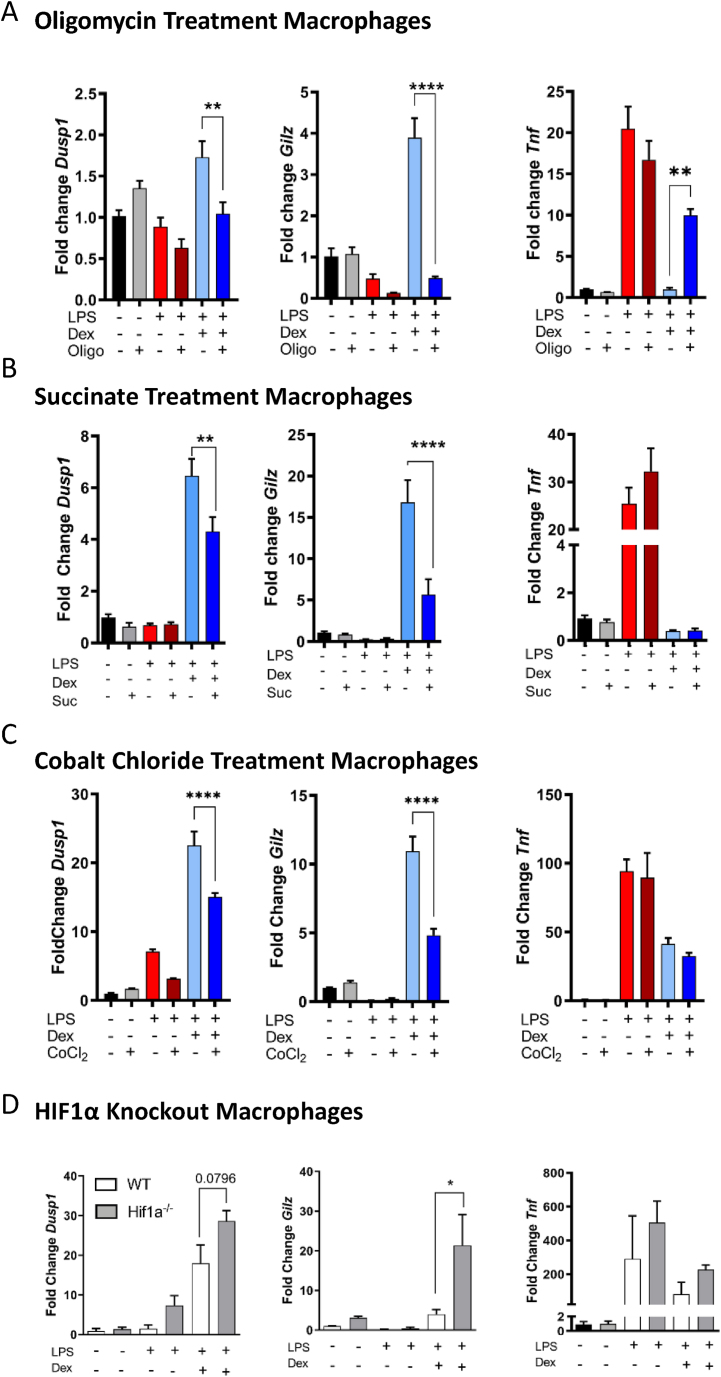
Tricarboxylic Acid Cycle Functionality and HIF1a Regulate Glucocorticoid Action. (A) Macrophages were pre-treated with oligomycin (5 μM) for 30 min (N = 5–6) or (B) 3 h with 5 mM succinate (N = 5–6) prior to incubation with vehicle, LPS (100 ng/ml) or LPS + Dex (100 nM) for 24 h and analyzed by RT-qPCR. (C) Macrophages were pretreated with CoCl_2_(100 μM) for 3 h before treatment with vehicle, LPS (100 ng/ml), or LPS + Dex (100 nM) for 8 h and analyzed by qPCR (N = 5).(D) *Hif1a* knockout macrophages were treated with vehicle, LPS, or LPS + Dex for 8 h before analysis by RT-qPCR (N = 3–4). Data are shown as mean + SEM. Statistical analysis was performed by two-tailed T-test (paired comparison) or one-way ANOVA with a Tukey post-hoc test (multiple comparisons). p < 0.05∗, <0.01∗∗, <0.001∗∗∗, <0.0001∗∗∗∗.

Previous reports have identified that while the anti-inflammatory signaling molecule interleukin-4 rewires macrophage metabolism, these alterations are also important for interleukin-4 functionality [30]. We therefore hypothesized that GCs not only alter macrophage metabolism but also require metabolic regulation for effectiveness of GR-mediated changes in transcription. We used the ATPase complex V inhibitor, oligomycin, to dissect out these effects. Treatment with oligomycin decreases oxidative phosphorylation and increases glycolysis as cells respond to the energetic demand, preventing TCA cycle progression through the accumulation of NADH+ [31]. Macrophages were pretreated with oligomycin for 30 min and then with either vehicle, LPS, or LPS + Dex for a further 24 h. We then quantified the key anti-inflammatory genes upregulated by GCs (*Gilz* (*Tsc22d3*) and *Dusp1*) [8] (Figure 5A) and observed that oligomycin treatment completely inhibited the Dex-mediated upregulation of *Gilz* and *Dusp1*. Repression of the pro-inflammatory cytokine *Tnf* by GCs was also abrogated by oligomycin treatment (Figure 5A). These effects, however, were not dependent either on oligomycin regulation of GRmRNA expression or on GR protein abundance (Figs. S5A and B).

This indicates afunctional control of GR-dependent gene regulation by mitochondrial metabolism and suggests that the effect of GCs on macrophage metabolism is necessary for their anti-inflammatory activities. This reliance on mitochondrial activity was also observed in dendritic cells, albeit to a lesser extent (Fig. S5C). We next pretreated macrophages with the cell-permeable diethyl-succinate to bypass the preventive effect of GCs on succinate accumulation, before incubation with vehicle, LPS, or LPS + Dex, and analyzed gene expression (Figure 5B). In a similar manner to ATPase inhibition, succinate attenuated the upregulation of the anti-inflammatory genes *Dusp1* and *Gilz*, which again was not dependent on the GR expression (Figs. S5A and B). However, succinate had no effect on GC-mediated repression of *Tnf*, indicating a specific effect of succinate on gene activation, unlike the effect of oligomycin.

### 2.7 HIF1α abrogate glucocorticoid actions in myeloid cells

Glycolysis and the inflammatory Warburg effect in macrophages are driven, in part, by succinate-dependent HIF1α expression (23). As we identified that GCs promote TCA cycle anaplerosis at the glutamine-α-ketoglutarate pathway, prevent succinate accumulation, and regulate ROS production, we hypothesized that GCs could inhibit the LPS-induced expression of HIF1α to promote succinate metabolism and inhibit glycolysis. GCs suppressed *Hif1a* mRNA (Figure 1C) and reduced the expression of HIF1α protein (Fig. S5D). We also found a significant reduction of nuclear HIF1α in peritoneal macrophages isolated from GR^flox^ mice treated with LPS + Dex compared with their LPS-treated counterparts (Fig. S5E). There was also a small, but significant, reduction in macrophage nuclear HIF1α in GR^LysMCre^ mice treated with Dex, likely due to the known limitations of the LysM-CRE, which have around a 70% recombination rate in macrophages [14]. However, the Dex-treated GR^LysMCre^ macrophages had significantly more nuclearHIF1α in comparison with the Dex-treated GR^flox^ cells (Fig. S5E), suggesting increased HIF1α activity. As a readout of HIF1α activity, we probed the RNA-seq in Figure 1 for HIF1α target genes based off the HIF1α KEGG pathway. Indeed, multiple genes in the HIF1α pathway were also regulated by LPS + dex, suggesting reduced HIF1α activity, in the LPS + dex-treated macrophages compared with LPS alone (Fig. S5F).

As LPS induces HIF1α accumulation [23], and GCs downregulate HIF1α, we used a pre-treatment with CoCl_2_ to artificially increase cellular HIF1α expression, followed by treatment of macrophage with vehicle, LPS, or LPS + Dex (Figure 5C). Indeed, CoCl_2_ inhibited the GC-dependent regulation of the anti-inflammatory genes *Gilz* and *Dusp1*, but not the repression of *Tnf*, and with no effect on GR abundance (Figs. S5A and B). Next, we used macrophages with a genetic deletion of *Hif1a* to determine the contribution of HIF1α to GC regulation of gene expression. Both wildtype and *Hif1a*^*−/−*^macrophages were treated with vehicle, LPS, or LPS + Dex (Figure 5D). Indeed, upon *Hif1a* deletion, there was an upregulation of *Gilz* and*Dusp1*in response to Dex under LPS-treated conditions compared with the wildtype cells. This suggests that without HIF1α, GCs are more capable of inducing an anti-inflammatory macrophage response and that downregulation of *Hif1a* is a core aspect of GC anti-inflammatory activities in macrophages. The effect, however, was independent of cytokine regulation as the repressive effect of Dex on *Tnf* gene expression was unchanged between the genotypes, matching the data on succinate pretreatment, and artificial HIF1α stabilization.

Taken together, these findings demonstrate that mitochondrial function is needed for GR regulation of pro- and anti-inflammatory genes in myeloid cells. While GCs did not increase oxidative phosphorylation *per se*, GCs inhibited the LPS-induced glycolytic switch in macrophages and dendritic cells. GCs also corrected the inhibitory effects of LPS on the TCA cycle, reducing succinate and ROS accumulation. By preventing the transcriptional and post-translational effects of LPS onHIF1α, GCs force macrophages away from glycolysis and ROS production. We also provide evidence that HIF1α is an important regulator of GR function in macrophages, and that hyperactivation of HIF1α inhibits GC-mediated gene expression, while its deletion promotes GC-mediated gene expression.

## 3 Discussion

GCs have long been known to regulate metabolism on an organismal level, and more recently on a cellular level (17, 18, 32); however, this has not been directly linked to the major role of GCs as anti-inflammatory compounds in immune cells. Here, we show for the first time that GCs rewire macrophage metabolism during inflammation. We also demonstrate that coordination of succinate metabolism in important for the anti-inflammatory effects of GCs. Pharmacological doses of GCs are capable of altering the mitochondrial network of macrophages both *in vitro* and *in vivo*, as well as regulating a subset of metabolic genes and metabolites associated with glutathione and succinate metabolism. GCs further increased TCA cycle anaplerosis between glutamine and α-ketoglutarate while having very little effect on the oxidative capacity of macrophages. GCs did however suppress inflammatory glycolysis. We further linked GC-regulated macrophage metabolism to gene regulation, demonstrating that with severe mitochondrial inhibition, or excess succinate, GC-gene activation was diminished. Furthermore, this effect is dependent on HIF1α, an important component of the cellular metabolism network. These data cement GR within the intracellular metabolism network, which regulates macrophage function during inflammation.

Global profiling of genes and metabolites highlighted GC regulation of macrophage immunometabolism. While the effect of GCs on inflammatory processes was expected, the striking result was the identification of metabolic pathways as enriched, especially those associated with mitochondria. Previous expression profiles also suggested regulation of metabolic traits in macrophages by GCs [7,33]; however we linked our findings to the consequences of metabolite regulation and glucose metabolism. Our integrative analysis clarified that the gene expression and metabolite abundance changes converged on pathways associated with ROS buffering and the TCA cycle. ROS production contributes to bactericidal effects in macrophages but is also a major driver of tissue damage in chronic or excessive inflammation [34]. GCs therefore control TCA cycle flux and regulate metabolites involved in the glutathione and lysine degradation pathways, all contributing to GC-driven tissue protection from damage and resolution of inflammation. We chose to focus on succinate metabolism as a major focal point, due to its well-known role in macrophage immunometabolism [[23], [35], [36], [37]]. Succinate can not only stabilize HIF1α but also alter the activity of other proteins through succinylation of lysine residues, malking a case for HIF1α-independent effects of succinate on GR-mediated gene regulation. This can affect the enzymatic functions of the modified proteins, contributing to effects on cell metabolism [34]. Competition between succinate and α-ketoglutarate can also inhibit histone demethylases, linking succinate accumulation to epigenetic control of gene regulation [35]. While we only assessed intracellular succinate, the release of succinate into the extracellular space can act paracrine via the succinate receptor (SUCNR1). Of note, SUCNR1 is expressed in metabolic organs such as the liver and white adipose tissue, and succinate metabolism has been linked to global glucose and lipid homeostasis [39]. Thus, future directions should focus on understanding how GC control of the TCA cycle can influence not only macrophage metabolism but also whole-body metabolism, whether acting locally via macrophages or by signaling to other cell types. In addition, it would be necessary to untangle the HIF1α-dependent effects and independent effects of GC control of succinate metabolism.

As mentioned above, the regulation of TCA cycle succinate but also glutathione/ROS metabolism by GCs readily links GC activity to the stabilization of HIF1α(26), previously unappreciated in macrophages. ROS in combination with TCA cycle disruption and succinate accumulation [23] results in the activation of HIF1α signaling in pro-inflammatory macrophages, which is further amplified by the inhibitory effect LPS has on SDH activity [22]. Indeed, GCs suppressed both the protein and RNA expression of HIF1α, along with HIF1α target genes and glycolytic metabolism of macrophages, a major HIF1α effect. But furthermore, they also increased SDH activity, which in part counteracts the disturbance of the TCA cycle. We also identified HIF1α as a regulator of GR action, whereby excess stabilization using CoCl_2_or succinate inhibited GR-mediated gene expression. Mirroring this effect, knockout of HIF1α promoted GR action in macrophages. There have been multiple studies linking GC signaling to HIF1α, albeit not in macrophages. Surprisingly, the mechanisms vary depending on the cell type observed [[40], [41]].

Recently, cistromic studies in HELA cells have demonstrated that hypoxia can rewire the GR cistrome [[42], [43]]. In a similar manner to what we observed in macrophages, GILZ (TSC22D3) regulation by GCs was inhibited by HIF1α activation in HELA cells, opening the possibility of a conserved GR-HIF1α mechanism across classically activated macrophages and cancer cells, potentially linked by the Warburg effect and succinate metabolism. We therefore identified a new regulator loop in the macrophages between GR and HIF1α, and the reciprocal regulation between these two factors may serve to tune the macrophage metabolic flux dependent on tissue oxygenation.

The mitochondrial network and thus efficient mitochondrial metabolism in macrophages are important for the resolution of inflammation, promoting the development of anti-inflammatory functions and preventing excess inflammatory signaling [22,33]. While we saw no effects on the mitochondrial copy number, we did observe a significant reduction in the mitochondrial volume *in vitro*. This may be caused by shrinking of the mitochondria in response to LPS + Dex rather than a reduction in the absolute number of mitochondria. Shrinkage of mitochondria can be caused by changes in the redox homeostasis, which were highlighted in the metabolomic/transcriptomic integrative analysis, indicating glutathione metabolism [54]. Furthermore, GCs prevented the LPS-induced mitochondrial fragmentation; however, we did not find GC-mediated regulation of *Drp1, Opa1, Mf1,*or *Mf2*, the major regulators of mitochondrial fission and fusion [44], in our sequencing analysis. This may be an issue with kinetics, and these genes may be acutely regulated by GCs rather after 24 h, or GCs may alter their activity rather than expression. Efficient mitochondrial metabolism, and thus TCA cycle anaplerosis allows the clearance of inflammatory intermediates that build up under LPS-treated conditions, especially succinate [35], which control multiple downstream inflammatory processes. Indeed, promoting this metabolic phenotype *in vivo* using Dex recapitulated some of the effects we see *in vitro*. Treatment with Dex had a protective effect on core temperature in GR^flox^ mice, indicating the necessity of macrophage GR responses for disease tolerance during endotoxin shock. Importantly, the effects we saw were dependent on GR expression itself, in the absence of GR in macrophages, the cells were unable to regulate the mitochondrial network, and metabolic genes in response to Dex. Due to the long duration of treatment, both *in vitro* and *in vivo*, we cannot determine whether the effects of GCs on metabolic function are through direct GR target genes, or secondary effects altered by GR-regulated genes. This should be further investigated in future studies; however, due to the lack of regulation of oxidative phosphorylation, which occurs in IL-4-mediated metabolic control, we hypothesize that GR acts independently of IL4 signaling [20, 22, 31, 46]. GCs were able to regulate TCA cycle anaplerosis at the glutamine-α-ketoglutarate pathway and the mitochondrial network independently from oxidative phosphorylation, which suggests that GCs drive an alternate program of metabolic rewiring in macrophages. This alternate program is entirely different from the well-studied IL-4-dependent effects [20,22,30,45], despite having a rather similar – anti-inflammatory – outcome.

Multiple TCA cycle components are capable of altering gene regulation through epigenetic means. For example, acetyl-CoA, the entry point of the TCA cycle is the major donor for histone acetylation (28), thus providing a direct link from cellular metabolism to gene regulation. Indeed, modulating macrophage metabolism using oligomycin drastically inhibited GC regulation of the core GR target and anti-inflammatory genes Gilz and Dusp1, providing a proof of principle that GR requires efficient mitochondrial function for access to certain genes. Also, LPS itself causes changes in mitochondrial function [22], suggesting that inflammatory conditions could paradoxically inhibit GR action. Recently, in a model of polymicrobial sepsis, the ability for GR to regulate genes in the liver was substantially limited during inflammation [47], indicating a conserved mechanism across cell types. Whether LPS mitochondrial dysfunction stratified cells into responders or non-responders at the single-cell level should be investigated in more detail.

This study provides the basis for GC regulation of myeloid immunometabolism, linking GC anti-inflammatory actions to mitochondrial function, through the regulation of glutathione/ROS production and TCA cycle functionality. We also identify a HIF1α-dependent mechanism of inhibition of GC signaling during inflammatory activation of macrophages. We propose that the regulation of cellular metabolism is therefore a key aspect of GC-mediated anti-inflammatory functions and may be used to find further strategies to limit excessive inflammation in a clinical setting.

## 4 Methods

### 4.1 Animals

All animal experiments were performed in accordance with the accepted standards of animal welfare and with permission of the responsible authorities of the Regierungspräsidium Tübingen, license 1332. Myeloid-specific GR mutant mice (*Nr3c1*^*tm2GscLyz2tm1(cre)lfo/J*^), GR^LysMCre^, on a C57BL/6 background were described previously [14]. Myeloid-specific HIF1α mutant mice (or *Hif1a*^tm3Rsjo^; Lyz2^tm1(cre)Ifo^), Hif1a^LysMCre^, on a C57BL/6 background and wildtype controls were used to generate primary cells. Mixed-sex cohorts between 8 and 15 weeks old were used for all the experiments.

### 4.2 Mouse model of endotoxin shock

Mixed-sex cohorts of mice aged between 10 and 14 weeks were injected i. p. with either vehicle (PBS) or LPS (Sigma–Aldrich, L2880) at a concentration of 10 mg/kg. The mice were then injected i. p. with either 1 mg/kg Dex (Sigma–Aldrich, D2915) or vehicle control. The mice were killed 24 h later. Mice body temperature and body mass were measured every 4 h after LPS injection. The mice were sacrificed if limit points were reached, i.e., body temperature lower than 28 °C and a total body weight loss of 15%. Sickness-related behaviors were also considered.

### 4.3 Isolation of peritoneal macrophages

Peritoneal cavity of mice was washed with RMPI-1640 (Sigma, R7388) and lavage was centrifuged at 300 g for 5 min. The resulting pellet was incubated in RPMI-1640 supplemented with 10% FCS (Sigma) for 3 h at 37 °C and 5% CO_2_. Non-adherent cells were then washed off with PBS, and the remaining cells were fixed with 4% *para*-formaldehyde before downstream analysis.

### 4.4 Isolation of lung macrophages

Lungs were harvested, minced, and digested using collagenase (Gibco, 17,018–029) for 1 h at 37 °C. The resulting suspensions were filtered through a 70 μm cell strainer and sorted using an Aria III system (BD Biosciences).

### 4.5 Cell culture

BMDMs were prepared as described previously [13], however using 30% L929 conditioned medium, and maintained for 7 days at 37 °C and 5% CO_2_. The cells were washed with PBS and maintained in macrophage serum-free medium (Macrophage SFM, Thermofisher) overnight before all experiments.

BMDCs were produced by flushing the femur and tibia and maintaining the resulting bone marrow in macrophage serum-free medium, containing 10% fetal calf serum (Sigma), 1% penicillin/streptomycin, 1% l-glutamine, and 50 μM β-mercaptoethanol. The cells were seeded at 2 × 10^6^ cells per 10 cm plate and supplemented with 20 ng/ml GM-CSF (Sigma Aldrich, SRP3201) to initiate differentiation. After 3 days of culture at 37 °C and 5% CO_2_, an additional 10 ml of medium with 20 ng/ml GM-CSF was gently added into the dishes. After another 3 days of differentiation, 10 ml of medium was removed, centrifuged for 5 min at 1500 rpm, resuspended in 10 ml of medium with 20 ng/ml GM-CSF, and added back into the culture. After 2 more days, the cells were harvested.

All cells were treated with vehicle (DMSO), Dex (100 nM), LPS (100 ng/ml) (all Sigma, Germany), or a combination of LPS + Dex for 24 h unless otherwise stated. Oligomycin (5 μM) (Sigma, Germany) was used for 30 min pretreatment before the addition of LPS or LPS + Dex. Diethyl succinate (5 mM) (Sigma, Germany) was used for 3 h pretreatment. Cobalt chloride (100 μM) was used for a 3-h pretreatment.

### 4.6 Reactive oxygen species detection

Cells were plated at 200,000 cells per well and treated with vehicle, LPS, or LPS + Dex for 16 h. The cells were then washed 3 times with PBS and incubated with 2′,7′-Dichlorfluorescein-Diacetate (DCFDA) (Sigma, Germany) diluted in PBS at 20 μM for 10 minat 37 °C, before washing with PBS and measuring on a fluorescent plate reader (CLARIOstar PLUS, Germany). The average MFI of technical duplicates was used for each data point.

### 4.7 RNA isolation and RT-qPCR

RNA was isolated using Trizol (Invitrogen), and equivalent concentrations of RNA in each sample were reverse transcribed using High-Capacity cDNA Kit RT (ThermoFisher). Real-time PCR was performed with SYBR Green PCR Master Mix (Invitrogen) using a ViiA 7 (Applied Biosystems) and analyzed using the delta–delta CT method.

### 4.8 RNA sequencing analysis

Sequencing was performed using the Novoseq platform (Novogene, China) and differentially expressed genes between LPS and LPS + Dex (p < 0.05) were subjected to gene ontology analysis using ENRICHR [55]. The genes were selected for ontology terms related to metabolism and clustered. The clusters were re-analyzed individually by ENRICHR. Clustering was performed using R software. Data can be found at GSE167382.

### 4.9 Metabolomics analysis

11 × 10^6^ BMDMs were treated for 24 h before washing 3 times with cold PBS and pelleted before snap freezing. Samples were analyzed using the BGI platform (BGI, Hongkong). In brief, 25 mg of cell pellet was homogenized and the metabolites were extracted for LC-MS analysis. QC was performed on an equal amount of all samples pooled together. The metabolites were separated and detected using a Waters 2D UPLC (Waters, USA) and a tandem Q-Exactive high-resolutionmass spectrometer (Thermo Fisher Scientific, USA). Analysis was performed with metaX [49]. Partial Least Squares Method-Discriminant Analysis (PLSDA) and Variable Importance in Projection (VIP) values of the first two principal components of the model, combined with variability analysis, were used to generate the fold change and theStudent'st-test was used to screen for differential metabolites. Pathway analysis was performed with MetaboAnalyst v5.0 [[45], [48]]. Clustering was performed using R software.

### 4.10 Seahorse Extracellular Flux analysis

Cells were treated with vehicle, Dex, LPS (10 ng/ml and 100 ng/ml) or a combination and analyzed by Seahorse assay as described previously [50], with minor modifications (60,000 cells/well, oligomycin (2 μM), FCCP (2 μM) rotenone (0.5 μM), antimycin A (0.5 μM) initial injection with glucose (10 mM)). Data were normalized to DNA content per well using picogreen staining (Invitrogen) and analyzed on a Tecan multimode reader (Tecan, Switzerland).

### 4.11 Metabolic Flux by Gas Chromatography and Mass Spectrometry

BMDMs pooled from 7 mice were pretreated with vehicle, LPS, or LPS + Dex in triplicate for 24 h before incubation for 2 h in 1 ml of RPMI-1640 (Sigma) at 37 °C in a closed tube with either 0.9 mg/ml U–^13^C_6_ glucose (Campro Scientific, Germany), 1,2–^13^C glucose, or 0.6 mg/ml U–^13^C5 glutamine (Cambridge Isotope Laboratories, USA). Unlabeled glucose and glutamine were added for a final concentration of 1.8 mg/ml and 0.6 mg/ml, respectively. Molecular ions as well as fragments of aspartate, glutamate, lactate, and itaconate were measured by GC/MS (5975–6890, Agilent, Waldbronn), and their carbon labelling patterns were analyzed to estimate TCA or pentose phosphate shunt flow rates with samples run in duplicate. For the analysis of metabolic flux data, we used RStan (R interface to Stan) a library for Bayesian modelling, which utilizes user-defined models and data to return posterior simulations of prior defined parameters [[51], [52]],to obtain absolute flow rates from the cumulative release of ^13^CO_2_, itaconate, and lactate. Data were converted to Z-scores, and the relative rates of each condition were displayed.

### 4.12 Succinate measurements

Intracellular succinate was measured using a succinate assay/measurement kit (abcam ab204718) according to the manufacturer's instructions

### 4.13 MTT assay

A total of 60,000 BMDMs were seeded per well of a 96-well plate. The cells were treated for 24 h, as indicated above before the addition of 3-(4,5-dimethylthiazol-2-yl)-2,5-diphenyltetrazolium bromide (MTT) (Roche 11,465,007,001) at 0.5 mg/ml for 4 h at 37 °C and 5% CO_2_. MTT was dissolved using DMSO, and the absorbance was measured at 570 nm.

### 4.14 Immunofluorescence and Imaging

Cells were plated at 500,000 cells/well onto coverslips and left to adhere overnight before serum starvation the following day. The cells were treated with vehicle (DMSO), Dex (100 nM), LPS (100 ng/ml) (all Sigma, Germany), or a combination of LPS + Dex for 24 h before fixation with 4% PFA for 1 h at 4 °C and consecutively washed with PBS before permeabilization with 0.1% TritionX-100 (Sigma) for 30 min at 4 °C. After washing with PBS, the cells were blocked with 0.5% FCS overnight at 4 °C.The next day, the cells were then stained with anti-TOM20 (1/200, D8T4N, Cell Signaling) overnight. On the next day, secondary anti-rabbit Alexafluor-488 (1/500, 21,206, Thermofisher) was used to detect the primary antibody. After washing, the cells were stained with DAPI and mounted using Fluromount (Sigma) and imaged with a Leica TCS SP8 microscope to acquire z-stacks of cells. Images were deconvolved and analyzed using the Mitochondrial AnalzyerImageJ plugin [53].

### 4.15 Immunoblot analysis

Immunoblots were performed as described previously [16] with minor modifications. In brief, macrophage lysates were prepared by scraping cells into RIPA buffer (50 mM TrisHCl pH 7.4, 1% NP40, 0.25% sodium deoxycholate, 150 mM NaCl, 1 mM EDTA) with protease inhibitors (Roche, cOmplete Protease inhibitor cocktail) and phosphatase inhibitors (Roche, PhosSTOP). Protein (20 μg) was run on a 10% acrylamide gel and then transferred on ice for 1 h to a 0.22 μm nitrocellulose membrane (Santa Cruz sc-3718). The membrane was then blocked with 5% BSA (Serva, 11930.04) in TBS-T for 1 h before incubation with primary antibodies against HIF1α (Cell Signaling, D1S7W, 1/1000), GR (Cell Signaling D8H2, 1/1000), or β-actin (Sigma A1978, 1/2000). The primary antibodies were detected using goat anti-rabbit (Invitrogen, A10547) or goat anti-mouse (Dako-Agilent, AF3628) HRP-linked secondary antibodies (1/5000). Chemiluminescence was detected using Immobilon Forte Western HRP substrate (Merck Milipore, WBLUF0100) using a BioRadChemiDocMP.

### 4.16 Statistical analysis

Data were analyzed using Graphpad Prism version 9 (Graphpad). Individual comparisons were performed using a two-tailed t-test, and multiple comparisons were performed with one-way or three-way ANOVA with a Tukey post-hoc test. Details of the sample numbers can be found in the appropriate figure legend. N values represent data/cultures from individual mice unless otherwise stated.

## Author contributions

Conceptualization: G.C. and J.P.T. Methodology and investigation: G.C., U.S., E-M.W., S.V., M.H., F.Z., S.W., N.K., and D.T. Data analysis: G.C., U.S., E-M.W., J.V., and U.W. Resource acquisition: G.C., R.M., B.C., P.R., P.F–P., S.W., N.K., and J.P.T. Supervision: G.C. and J.P.T. Writing: G.C. and J.P.T wrote the manuscript. All authors edited the manuscript.
