# Supplementary figures and images for: Glucocorticoids coordinate macrophage metabolism through the regulation of the tricarboxylic acid cycle
Source: Mol Metab. 2021 Dec 22;57:101424. doi: 10.1016/j.molmet.2021.101424 (PMC8783148; doi:10.1016/j.molmet.2021.101424)

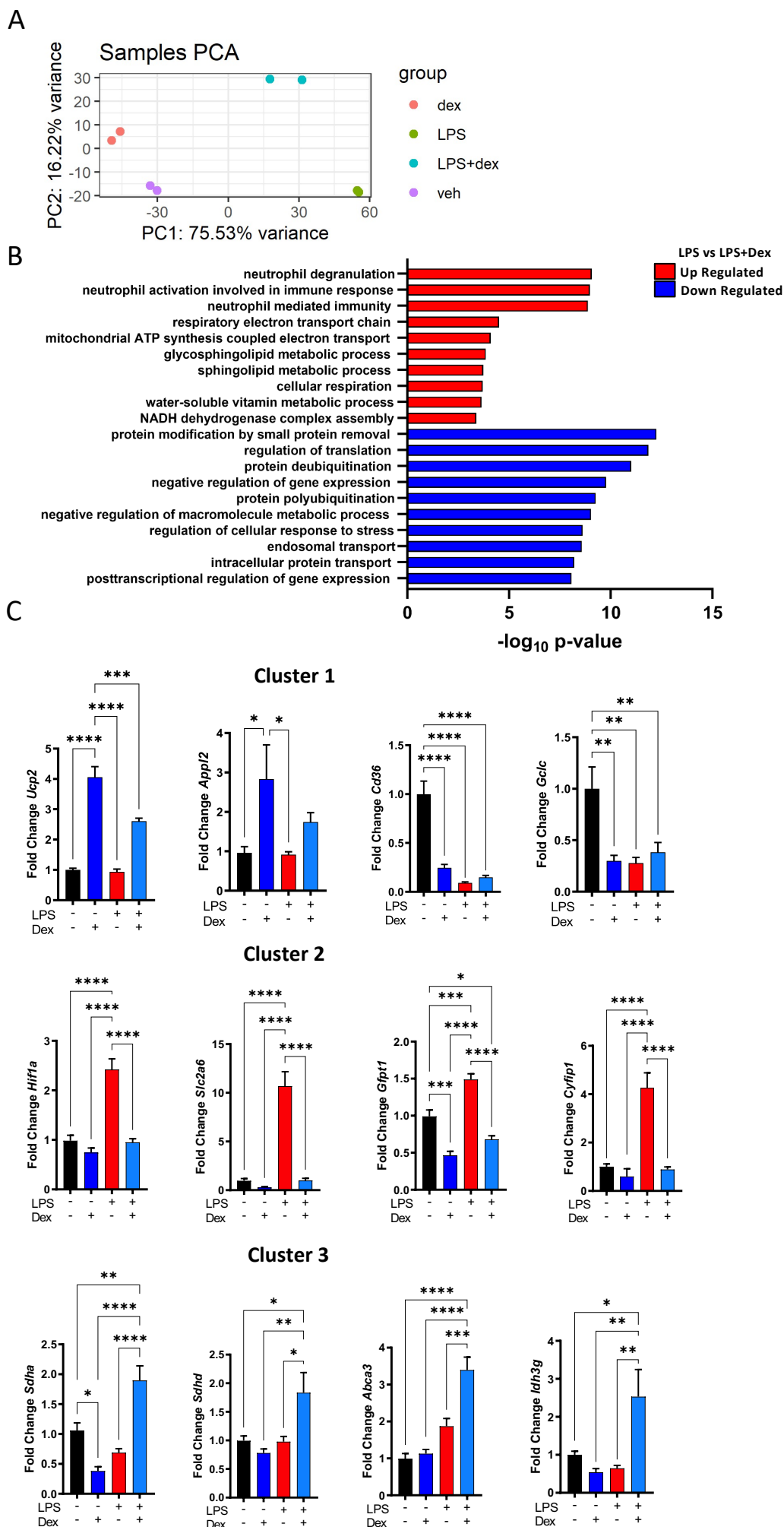

Figure. S1

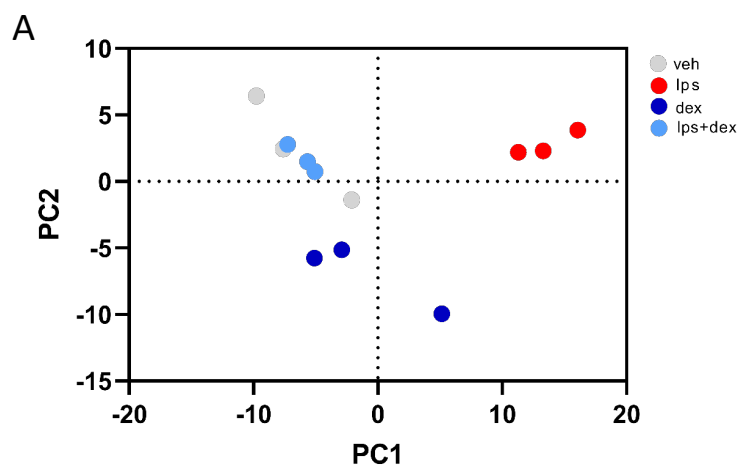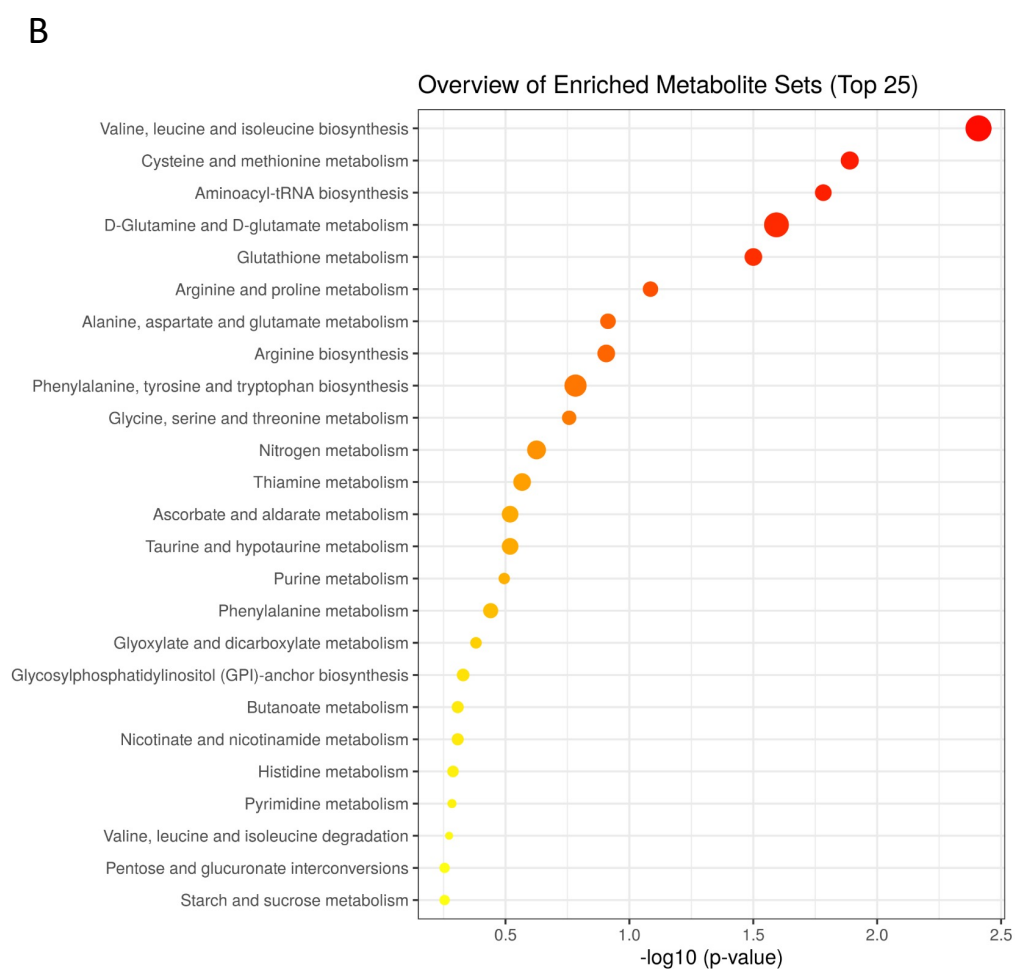

**C** Copy Number

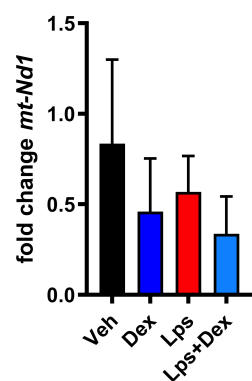

Figure. S2

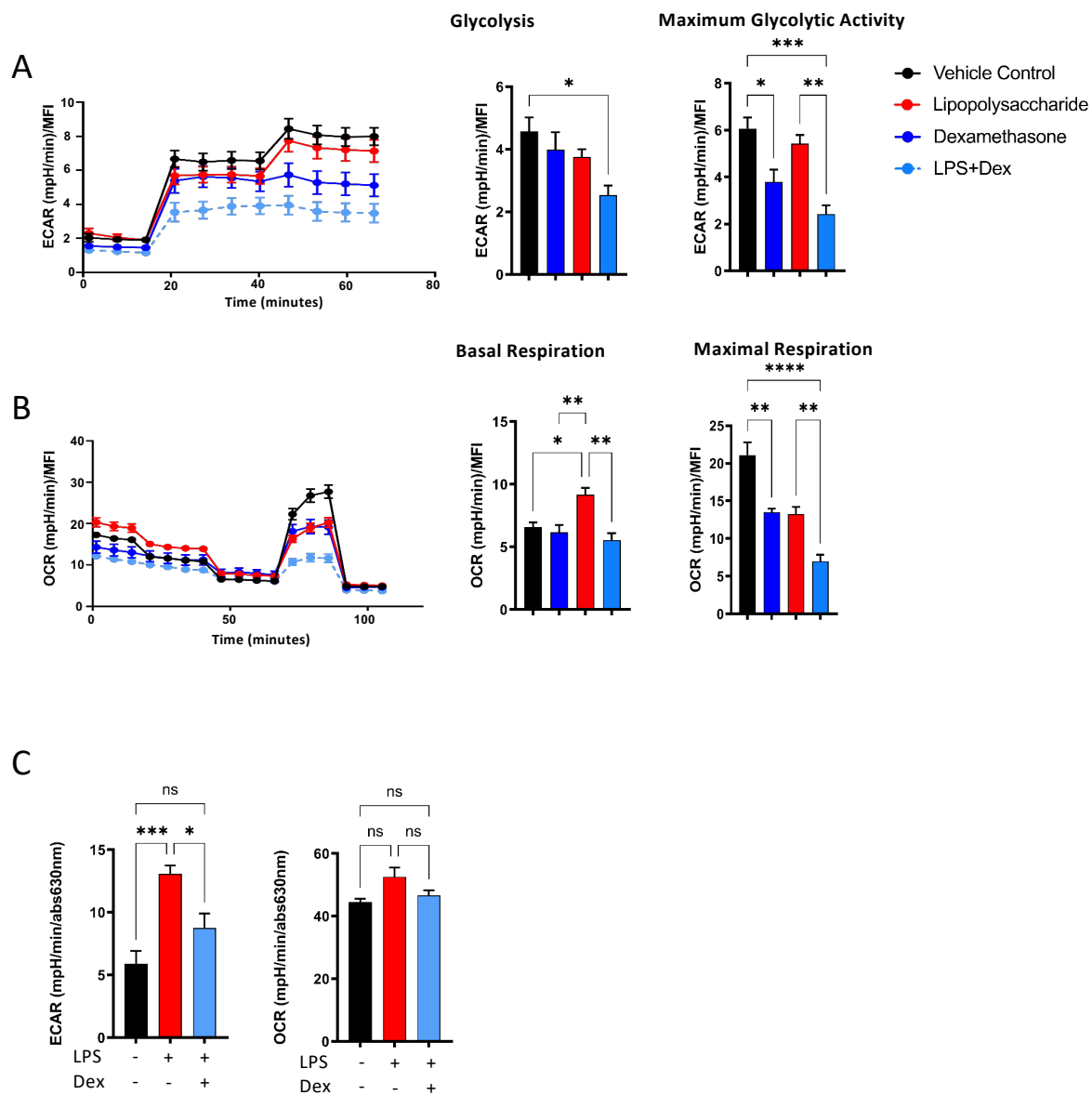

Figure. S3

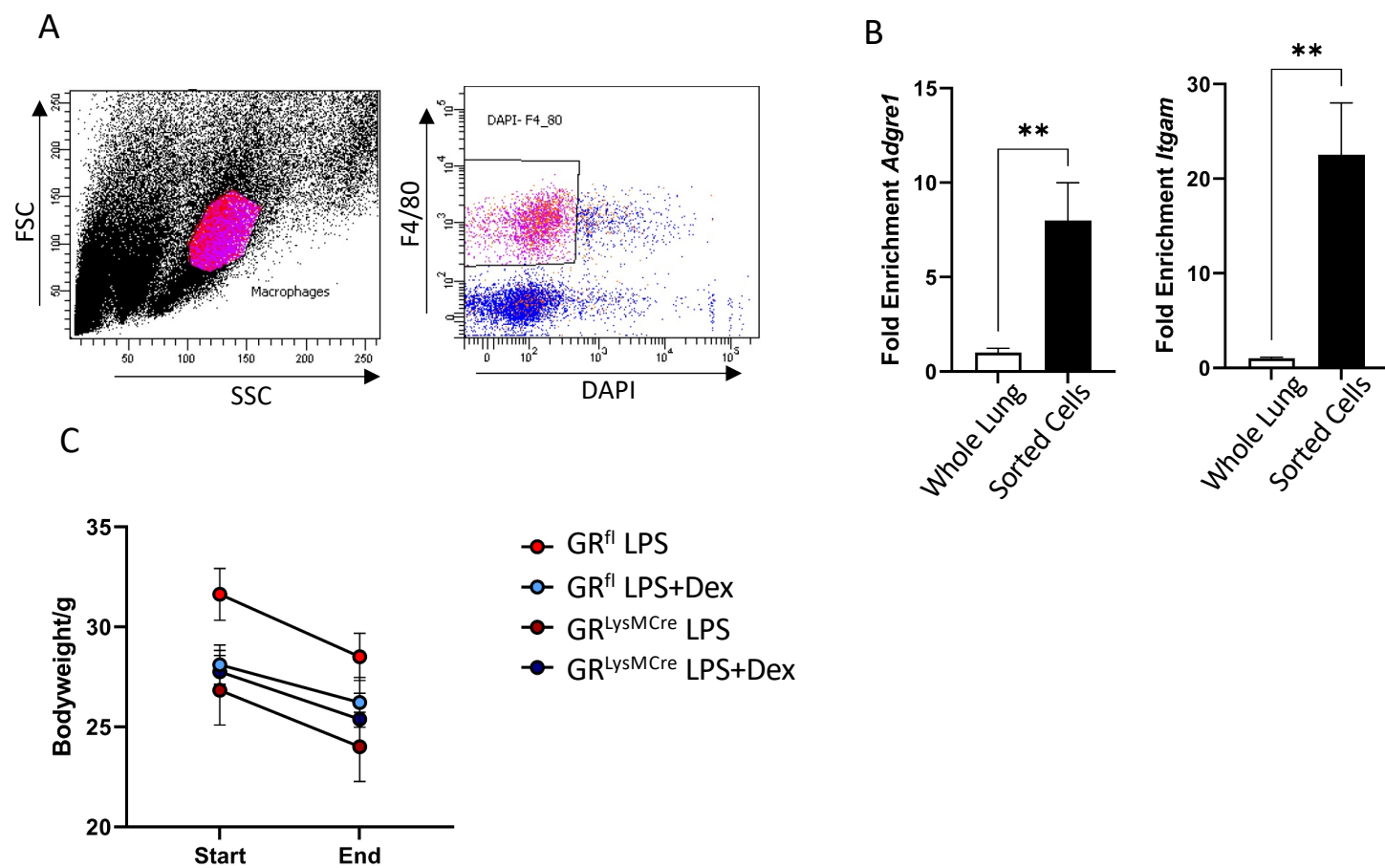

Figure. S4

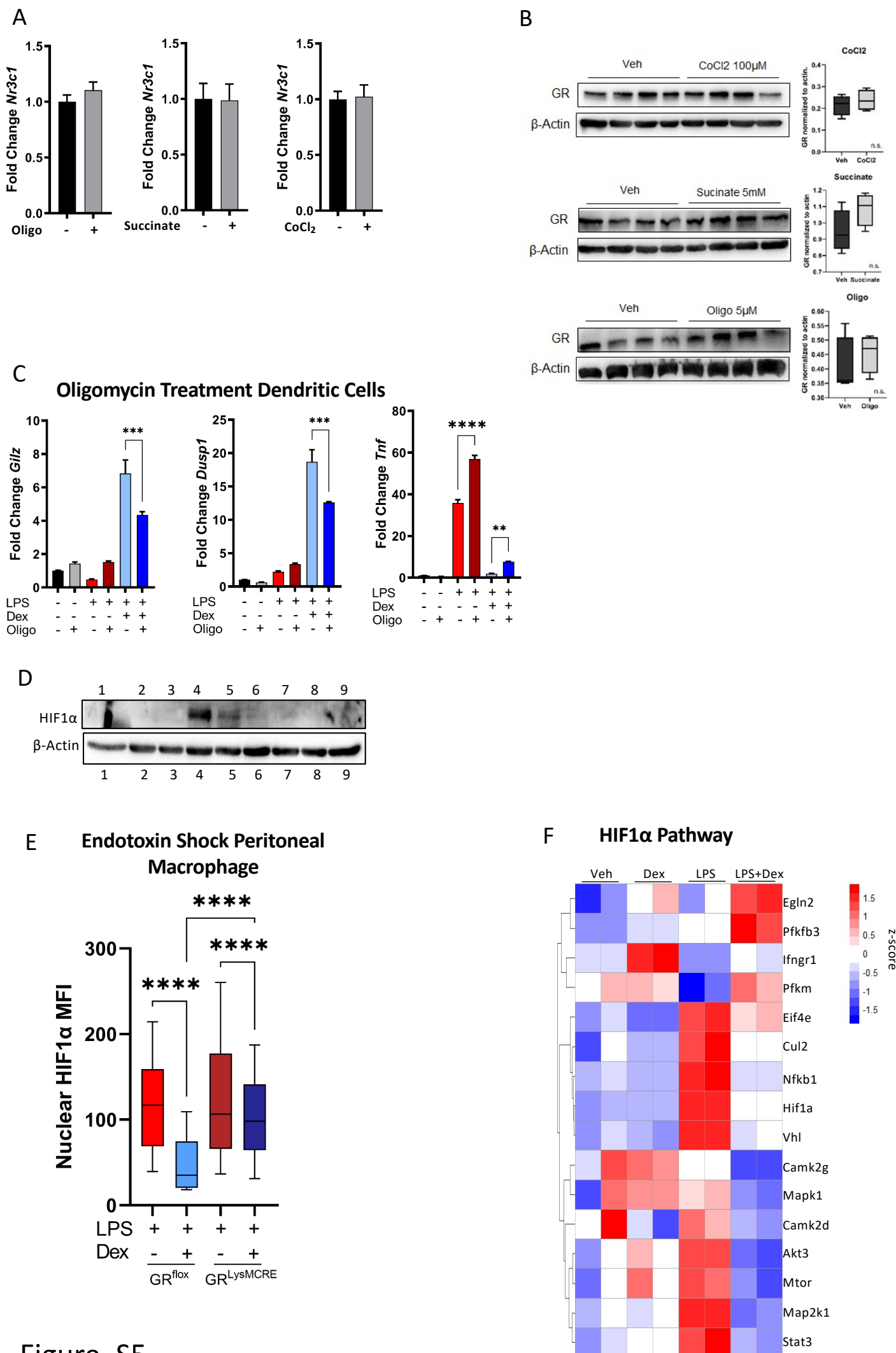

Figure. S5

Supplement: Supplementary file 4 — Multimedia component 4 [file mmc4.pdf]
